# A Review of Low-Density Lipoprotein-Lowering Diets in the Age of Anti-Sense Technology

**DOI:** 10.3390/nu15051249

**Published:** 2023-03-01

**Authors:** Paul J. Nestel, Trevor A. Mori

**Affiliations:** 1 Baker Heart & Diabetes Institute, Melbourne 3004, Australia; 2 Medical School, University of Western Australia, Perth 6000, Australia

**Keywords:** hypercholesterolemia, dietary approaches, cholesterol-lowering technologies

## Abstract

This narrative review discusses an important issue, the primary role of diet in reducing low-density lipoprotein cholesterol (LDLc) concentrations in polygenic hypercholesterolemia. Two effective drugs, statins, and ezetimibe, that lower LDLc > 20% are relatively inexpensive and potential competitors to strict dieting. Biochemical and genomic studies have shown that proprotein convertase subtilisin kexin type 9 (PCSK9) plays an important role in low-density lipoprotein (LDL) and lipid metabolism. Clinical trials have demonstrated that inhibitory monoclonal antibodies of PCSK9 dose-dependently lower LDLc up to 60%, with evidence of both regression and stabilization of coronary atherosclerosis and a reduction in cardiovascular risk. Recent approaches using RNA interference to achieve PCSK9 inhibition are currently undergoing clinical evaluation. The latter presents an attractive option of twice-yearly injections. They are, however, currently expensive and unsuitable for moderate hypercholesterolemia, which is largely due to inappropriate patterns of eating. The best dietary approach, the substitution of saturated fatty acids by polyunsaturated fatty acids at 5% energy, yields > 10% lowering of LDLc. Foods such as nuts and brans, especially within a prudent, plant-based diet low in saturates complemented by supplements such as phytosterols, have the potential to reduce LDLc further. A combination of such foods has been shown to lower LDLc by 20%. A nutritional approach requires backing from industry to develop and market LDLc-lowering products before pharmacology replaces the diet option. Energetic support from health professionals is vital.

## 1. Introduction

The purpose of this narrative review is to summarize information shown to improve the plasma lipid profile, in particular lowering LDLc, through diets and key dietary components, factors that, if not corrected, contribute to cardiometabolic disorders and cardiovascular risk. Pharmacological treatment is also required at times, especially when dyslipidemia is complex and likely to have a substantial genetic component. This area of research has yielded several highly effective drugs based on monoclonal antibody therapy and antisense technology, some key developments of which will be reviewed. While initially expensive and, therefore, likely to be used only in patients with significantly elevated risk, these novel pharmacological approaches will be more readily accessible as costs decline, as needs increase, and as methods of administration become more attractive than daily pills. The cost must be judged against an achievable 25% reduction in low-density lipoprotein cholesterol (LDLc) with monoclonal antibody therapy (leading to a reduction by about 50% in major cardiovascular events) and >90% LDLc reduction with RNA-silencing technology. The costs and benefits of these technologies will not be discussed since this is highly fluid.

## 2. Source of Evidence

We have searched articles published up to February 2023 derived from research involving human subjects, published in English, and indexed in MEDLINE (through PubMed), EMBASE, the Cochrane Library, and other selected databases. Key search words included, but were not limited to, “hypercholesterolemia”, “LDL-cholesterol”, “dietary approaches”, “cholesterol-lowering technologies”, “PCSK9”, and “clinical trials”.

## 3. LDL and Atherosclerotic Cardiovascular Disease

Dyslipidemias, including elevated plasma total cholesterol, LDLc or triglycerides, low plasma high-density lipoprotein cholesterol (HDLc), or a combination thereof, represent a major risk factor for ischemic heart disease [1]. According to a recent World Health Organization (WHO) report, the global prevalence of raised plasma total cholesterol among adults ≥25 years was approximately 39% in 2008 [2]. More than 30% of deaths from ischemic heart disease or ischemic stroke were attributable to raised plasma LDLc [3].

Numerous population and genetic studies have established the causality of LDLc in atherosclerotic cardiovascular disease [4]. A meta-analysis of 170,000 participants showed that the reduction in major vascular events with intensive lowering of LDLc is directly proportional to the absolute reduction in LDLc achieved [5]. Each 1.0 mmol/L reduction in LDLc is associated with an approximate one-fifth reduction in the annual rate of major vascular events, irrespective of baseline cholesterol [5]. These data suggest a 2–3 mmol/L reduction in LDLc would reduce risk by 40–50%. A recent position statement by Poli et al. [6] also addressed the issue of LDL cholesterol in the primary prevention of cardiovascular disease.

## 4. Evaluation of Diet on LDLc

### 4.1. Dietary Fat and Fatty Acids

This is not a historical review, but Ancel Keys [7,8,9] and Mark Hegsted [10] deserve credit and acknowledgment for research conducted in the 1950s and 1960s, which forms the basis of much that is known of the relative effects of different dietary fatty acids on LDLc. Their meticulous clinical nutrition studies allowed modeling that culminated in similar equations to quantify changes in total cholesterol levels as they occur in response to alterations in diet composition.

Keys’ equation [8]:

∆ total serum cholesterol (mg/dL) = 1.2 (2 ∆_SAT_ − ∆_PUFA_) + 1.5 √_Chol_ (1)

Hegsted’s equation [10]:

∆ total serum cholesterol (mg/dL) = 2.16 ∆_SAT_ − 1.65 ∆_PUFA_ + 0.068 ∆_Chol_ (2)

where ∆_SAT_ is the change in the percentage of dietary energy from saturated fats, ∆_PUFA_ is the change in the percentage of dietary energy from polyunsaturated fats, and ∆_Chol_ is the change in intake of dietary cholesterol (Keys: in mg/1000 cal/day; Hegsted in mg/day).

Both equations show that saturated fats increase total and LDLc twice as much as polyunsaturated fats lower them. A minor difference is that Hegsted et al. [10] reported that saturated fatty acids raise cholesterol levels somewhat less than reported by Keys et al. [8], whereas polyunsaturates lower them somewhat more. Keys and Hegsted determined that monounsaturated fatty acids have little or no effect on serum cholesterol in humans; Keys additionally determined that stearic acid and saturated fatty acids containing fewer than 12-carbon atoms have negligible effects on serum cholesterol.

It is worth noting that discussion of the effects of one fatty acid without considering the other has little meaning, that saturates exert a greater influence than polyunsaturates, and that the quantity of fat eaten may also be important. Hence, in populations where fat consumption contributes low amounts of energy, as in many countries, demonstrating that the nature of the fatty acids may or may not be a determinant of the LDLc concentration has limitations. That applies to some extent to the Prospective Urban Rural Epidemiology (PURE) study, which has received much publicity because of its conclusion that consumption of saturated fat is preferable to high carbohydrate intake to achieve lower cardiovascular risk (CVD). The PURE Study [11], a prospective cohort comprising 135,335 individuals from 18 countries (15, middle- and low-income), examined food consumption through questionnaires and mortality 7.4 years later. Mortality rates were lower with all three major classes of fats, including saturates, although the findings may have been influenced by the paucity of high-income countries. Saturated fat consumption was very low in China (5.7% energy) compared with 10.9% in Europe and North America, yet China comprised one-third of the study population. The study confirmed that if saturated fat intake is low, it is difficult to quantify its effect on mortality or cardiovascular outcomes.

The Keys and Hegsted equations predicted the limited effect of monounsaturates on total cholesterol concentration. This was confirmed much later in a study using liquid diets enriched with monounsaturated fatty acids. During three dietary periods, the study compared equicaloric diets that provided 40% fat energy and 43% carbohydrate as either monounsaturates (oleic acid (18:1*n*-9)-rich safflower oil) or saturates (mainly coconut oil), with a “low-fat” diet that had 20% fat and 63% I carbohydrates (glucose) [12]. Compared with the high-saturated fat diet, both high-monounsaturated and high-carbohydrate diets resulted in lower LDLc concentrations, −21% and −15%, respectively, which are substantial. However, the monounsaturated fatty acid palmitoleic, which is a constituent of palm oil, raises LDLc, unlike oleic acid [13,14].

A recent meta-analysis of 44 randomized controlled trials of isoenergetic replacement of palmitic acid (C16:0) with unsaturated fatty acids or oleic acid reported a mean reduction of LDLc by 0.36 mmol/L (95%CI: −0.50, −0.21 mmol/L) and 0.16 mmol/L (95%CI: −0.28, −0.03 mmol/L), respectively, or >10% in healthy individuals [15]. The PURE study confirmed some but not all of the accepted diets induced differences between lipid classes [11]. The claim that all three classes of fatty acids were associated with higher LDLc than with carbohydrates is correct, but the magnitude of the effects differed considerably. LDLc was highest with saturated fats and barely increased with mono- and polyunsaturates. The potentially undesirable effects of carbohydrate consumption included increased triglycerides and lower HDLc, in line with previous reports.

Clinical nutrition trials that directly compared foods enriched in either highly saturated or highly polyunsaturated fats while minimizing confounding from other nutrients have been performed using dairy and meat products from cattle and cows fed fatty acids protected from ruminal degradation to produce milk and meat differing in linoleic acid (18:2*n* − 6) content. In one such study, a randomized blinded crossover study of 33 participants, the mean LDLc was reduced by 0.24 mmol/L or about 10% in healthy individuals during the polyunsaturated food period [16]. Other technological manipulations that produced edible fats and oils with differing palmitic, oleic, and linoleic acid compositions in randomized clinical trials have also shown significant differences in LDLc between palmitic acid-enriched and unsaturated fatty acid-enriched food products [17]. Such blended and inter-esterified fats that are enriched in unsaturated fatty acids but retain enough hardness for baking requirements have entered conventional bakery markets and have made modest but favorable impacts on LDLc concentrations.

Not all saturated fatty acids raise LDLc, as has consistently been found for palmitic acid (C16:0). Stearic acid (C18:0) has consistently been shown to be “neutral” with respect to LDLc [18]. Similarly, saturated fatty acids in medium chain-length glycerides (C10-12:0) do not affect the LDLc concentration [19]. Short-chain saturated fatty acids (<C10) are not eaten to a significant degree but are formed in the colon during the degradation of carbohydrates, mainly fiber.

Uncertainty, however, prevails in relation to two other saturated fatty acids, myristic acid (C14:0), which is a major component in butter, and lauric acid (C12:0), a major component of tropical oils. In a study comparing palm oil (rich in palmitic acid), high-oleic acid sunflower oil, or a specially produced high-myristic acid fat, each providing about 10% of total energy, myristic acid raised LDLc significantly more than oleic acid (by 0.50 mmol/L) and even more than palmitic acid (by 0.11 mmol/L) [20]. Lauric acid (C12:0) is, by definition, a medium-chain saturated fatty acid; a synthetic form of lauric acid oil was shown to raise LDLc relative to sunflower oleic oil but not as much as palmitic acid in palm oil [21]. The major fatty acid in palm oil is palm olein, which has been shown to significantly raise LDLc to a similar extent as lard when compared with olive oil [22]. These findings have had commercial implications beyond nutritional considerations for the palm oil and dairy industries.

Dairy foods have, however, undergone reappraisal and have demonstrated that a single complex food group may be heterogeneous in the structure and composition of its products as well as its effects on LDLc. Milk fat is rich in saturated fatty acids, including myristic acid, the effect of which on LDLc concentrations has been controversial. As pointed out above, myristic acid has been shown to raise LDLc to a greater extent than palmitic acid. Similarly, equivalent amounts of milk fat in cheese and butter have differential effects on LDLc. Several clinical trials have shown that butter almost invariably elevated the LDlc concentration significantly more than cheese [23,24,25]. We have provided a more detailed discussion on aspects of dairy intake in relation to serum lipids and cardiovascular risk in a recent review [26].

Whether palm and coconut oils that are rich in saturates are likely to raise LDLc under all circumstances remains controversial. When consumed in amounts eaten in Western countries, both coconut and palm oil raise LDLc (7.8% and 4.6%, respectively) more compared with olive oil, suggesting that minimizing consumption of these oils is desirable [27,28]. When dietary fat is high, as in a study that compared coconut oil with butter and safflower oil at fat intakes of 50% energy, coconut oil consumption led to significantly higher LDLc than safflower oil, although less than when butter was tested [29]. However, when total fat consumption is low, as in many Asian nations, these oils are less likely to raise LDLc, which explains some of the controversy.

That *trans* fatty acids, derived from industrial hydrogenation, in amounts consumed in the past within hard margarines raise LDLc and lower HDLc is no longer disputed [30]. However, whether naturally occurring ruminant *trans* fatty acids are also potentially undesirable remains unresolved [31,32]. When eaten in amounts about equivalent to that known to raise LDLc from consuming hard margarines (3.7% energy in this study), ruminant *trans* fatty acids raise LDLc to a similar extent [33]. The virtual elimination of *trans* fatty acid isomers of oleic and linoleic acids from spreads has contributed significantly to healthier diets.

The particle size of LDL is considered to indicate CVD risk, with larger cholesterol-enriched particles associated with lower risk. Paradoxically, some studies have reported a predominance of larger circulating LDL particles with increased saturated fatty acid consumption [34,35].

There is considerable interindividual variability in responses to dietary fat manipulation, which is in part genetically determined. A study of single nucleotide polymorphisms (SNPs) identified 22 SNPs in genes related to lipid and bile acid metabolism on LDLc in response to 5 isoenergetic diets, two rich in saturated fatty acids from cheese or butter, compared with monounsaturated fatty acids, n-6 polyunsaturated fatty acids and a diet higher in carbohydrates [36]. After 4 weeks, endpoint LDLc levels (mmol/L) were as follows: cheese 3.18 ± 0.04, butter 3.31 ± 0.04, monounsaturates 3.00 ± 0.04, polyunsaturates 2.81 ± 0.04, and carbohydrates 3.11 ± 0.04. SNPs in two genes, *ABCA1-rs2066714* and apolipoprotein E isoforms, exhibited consistent significant influence.

Summarizing the effects of the major fats/oils in terms of their degree of saturation, Griffin et al. [37] calculated that isoenergetic replacement of 1% energy from dietary saturated fatty acids with polyunsaturated fatty acids (mainly linoleic) can be expected to lower LDLc concentrations by a mean of −0.055 mmol/L (95%CI: −0.061, −0.050). In an individual with LDLc of 3.0 mmol/L, a common signal for dietary intervention, fatty acid substitution represents a reduction of only 2%. This could be calculated to reduce cardiovascular risk by approximately 4%. Since dietary substitutions in practice are rarely less than 5% energy, the likely reduction in LDLc would be approximately 10%, which translates to an estimated 20%reduction in cardiovascular risk. This is in line with that calculated by epidemiologists for every 5% energy substitution [38]. The isoenergetic replacement of 1% energy from dietary saturated fatty acids with monounsaturated fatty acids (oleic) or carbohydrates was calculated to reduce LDLc to a lesser extent, −0.042 mmol/L (95%CI: −0.047, 0.37) and −0.033 mmol/L (95%CI: −0.039, −0.027), respectively.

### 4.2. Dietary Cholesterol

The evidence for restricting cholesterol-rich foods is less than that for saturated fat. Guidelines from Europe and the UK had been less restrictive than those from the USA. The 2019 American College of Cardiology (ACC)/American Heart Association (AHA) Guidelines on the Primary Prevention of Cardiovascular Disease became less prescriptive: “*A diet containing reduced amounts of cholesterol and sodium can be beneficial to decrease ASCVD risk*” [39]. The current 2019 European Society of Cardiology (ESC)/European Atherosclerosis Society (EAS) guideline [40] for reducing LDLc lists “*Reduce dietary cholesterol*” with a single + (contrasting with ++ to reduce dietary saturates).

Overall, meta-analyses confirm a clinically relevant association between cholesterol consumption and LDLc while acknowledging a lesser effect than that of dietary fats. The AHA Science Advisory published in 2020 [41] advises: ”*Patients with dyslipidemia, particularly those with diabetes mellitus or at risk for heart failure should be cautious consuming foods rich in cholesterol*”. Further, “*guidance focused on dietary patterns is more likely to promote diet quality and to promote cardiovascular health*”.

The heterogeneous effect of cholesterol among individuals is in part genetically determined and that individuals with elevated LDLc show the greatest response to dietary cholesterol is well known [42]. Meta-analyses and systematic reviews have shown modest nonlinear relationships between cholesterol consumption in foods, mainly eggs, and increments in LDLc [43,44]. The effect is nonlinear, diminishing above 900 mg consumption, equivalent to four eggs daily [45]. In randomized controlled trials, the increments in LDLc were greatest when eggs were the source of additional cholesterol [45]. Calculations on the effects on LDLc concentrations by reducing dietary cholesterol have been estimated: lowering cholesterol by 100 mg lowers LDLc by 4.5 mg/dL applying nonlinear models [43]. Since the average consumption is about 300 mg daily, it is apparent that lowering identifiable sources of cholesterol, mostly in eggs, will result in moderate reductions in LDLc but not as significantly as substituting saturated fatty acids with polyunsaturated fatty acids [46].

### 4.3. Other Dietary Approaches

In addition to dietary fats and oils, consuming other foods such as nuts, plant sterols, less readily digestible carbohydrates, and virtually indigestible fiber also lowers LDLc. Jenkins et al. [47] combined some of these foods in a “dietary portfolio of cholesterol-lowering foods” in a 1-yr trial in 66 hypercholesterolemic participants. Among full compliers (only one-third), LDLc was reduced by about 20% in response to the daily intake of plant sterols (1 g/1000 calories), soy protein (22.5 g/1000 calories), viscous fibers (10 g/1000 calories), and almonds (23 g/1000 calories). Although compliance was disappointing, these findings are consistent with other trials showing the benefits of consuming individually plant sterols and nuts. One conclusion from this trial was that the magnitude of LDLc achieved by the compliers approximated that found in trials with low-dose statins.

The above assertion has been tested In a recent single-blind clinical trial in which 190 adults with no history of atherosclerotic cardiovascular disease and an LDLc of 1.8–4.9 mmol/L were randomized to a low dose of a statin, rosuvastatin (5 mg), a placebo, plant sterols (1600 mg), fish oil (2400 mg), red yeast rice (2400 mg), garlic (5000 μg allicin), cinnamon (2400 mg), or turmeric curcumin with bioperin (4500 mg) [48]. Mean percent changes in LDLc from baseline were as follows: −37.9% for rosuvastatin, −2.6% placebo, −3.4% fish oil, +5.1% garlic, −4.4% plant sterol, and −6.6% red yeast rice (a compound that has statin-like properties). The change in LDLc after cinnamon and turmeric was negligible. To date, it is the only major head-to-head comparison between a low-dose statin and frequently recommended supplements for lowering cholesterol.

Other studies have reported better results for plant sterols at a similar dosage. Products containing plant sterols are predominantly in margarines, but milk, bread, and cereals have also been marketed, with each daily serving containing about 1.6 to 2 g of phytosterol. In a study of 58 men and women with mild hypercholesterolemia (6.2, SD ± 0.7 mmol/L), the consumption of 1.6 g/day of phytosterols as sterol esters in milk and yogurt for 3 weeks reduced LDLc by 15.9% and 8.6%, respectively, suggesting that incorporating phytosterol into dairy products may enhance efficacy [49]. Bread and cereal enriched with 1.6 g phytosterol resulted in significant but inferior LDLc lowering (6.5% and 5.4%, respectively) [49]. Clearly, phytosterol is a valuable supplement that is probably more efficacious when consumed within food.

Soy protein as a supplement or within appropriate foods has been promoted to reduce cholesterol concentration and has received approval from the U.S. Food and Drug Administration. However, efficacy has been questioned, and a meta-analysis of 41 controlled studies showed that soy protein at a median dose of 25 g/day during a median follow-up of 6 weeks resulted in a small, albeit significant 3–4% reduction in LDLc (0.12 mmol/L; 95%CI: −0.17, −0.07 mmol/L) [50].

Tree nuts have been consistently advised as part of total diets based partly on their LDLc-lowering effects. One systematic review, a meta-analysis of 61 controlled trials (2582 participants) with interventions ranging from 3 to 26 weeks, combined investigations of most commonly eaten nuts [51]. The study showed a modest 5% reduction in LDLc (−0.12 mmol/L; 95%CI: −0.14, –0.11 mmol/L). The dose response between nut intake and LDLc was nonlinear, with maximal benefit at 60 g/day.

Viscous fiber such as pectin, guar, etc. has been promoted for lowering plasma cholesterol, but the evidence is conflicting for both nondigestible and soluble fiber. Beta-glucan-rich brans from oats and barley have modest LDLc lowering effects, and each is of the order of 5% or less. One such comparison of large quantities (25 g/1000 calories) of soluble and nonsoluble fiber (authors’ terminology), with a low saturated fat, low cholesterol, and high in carbohydrate background diet, showed a 4.9 ± 0.9% fall in LDLc [52]. An equally unrealistic quantity of fiber (87 g/day) was compared with oat bran or refined wheat product during a 6-week intervention period, during which LDLc concentrations did not change significantly [53].

### 4.4. Total Diet

We have previously published impressive reductions in cardiovascular events with a variety of diets involving multiple approaches, including those reviewed above [54,55]. Optimizing body mass, increasing consumption of plant foods and fish, reducing salt and meat intake, and moderating alcohol consumption, as well as modifying the nature of dairy products advising consumption of yogurt, summarize the whole-diet approach.

Similar dietary modifications are the basis of the National Cholesterol Education Program (NCEP), which has Step 1 and Step 2, the latter of which is more stringent, being low in total fat (18–29% energy) and in saturated fatty acid (4–7% energy) [56]. The degree of variation in response by both men and women was large, demonstrating that real-life experience differs from that in randomized controlled trials. The changes in LDLc ranged from +3 to −55% in men and from +13 to −39% in women. A large component of the variability in LDLc response to the diet was accounted for by age and the concentration of LDLc at baseline (48%) in men and by age (13%) in women.

### 4.5. Implications for HDL and Triglyceride Concentrations

The following summarizes only the key points since the primary focus of this review is to ascertain whether diet still has a leading role in managing elevated LDLc, given the availability of powerful drugs that diet cannot match. In modifying diets, it is self-evidently necessary to minimize adverse effects on the whole plasma lipid profile. Fats and saturates, in particular, in general raise high-density lipoprotein (HDL) cholesterol (HDLc), but this is no longer seen as significantly advantageous; HDL is part of the cholesterol transport system, which is regulated at multiple points [57]. The enzyme cholesteryl ester transport protein (CETP) transfers cholesterol from HDL to LDL [57]; its activity is increased when *trans* fatty acids are consumed, leading to lower HDLc and higher LDLc [58].

In contrast with the downgrading of HDL as an independent cardiovascular risk factor, a multiple single nucleotide polymorphism (SNP) Mendelian randomization analysis in over 62,000 participants has shown that plasma triglyceride concentrations causally increase the risk of coronary heart disease (CHD) [59]. It becomes important not to raise the triglyceride level through dietary interventions that target LDLc. The main risk occurs when fat intake is reduced substantially and replaced by readily digestible carbohydrates. The amount of carbohydrate becomes a factor when it exceeds about 60% of total energy intake. It is preferable to replace saturated fatty acids with more unsaturated fatty acids and maintain carbohydrates at <55% energy. A major long-term prospective study has shown optimal carbohydrate consumption to be 50–55% energy [60].

Other key recommendations for reducing raised triglyceride levels are not controversial: maintaining normal body mass or as near to that as possible, exercising regularly, drinking alcohol moderately according to national standards, and eating fish regularly, including oily varieties [61,62]. Whereas weight loss in overweight hypertriglyceridemic individuals can be highly effective by substantial lowering of plasma triglyceride levels, weight loss has variable and at most modest LDLc-lowering efficacy irrespective of the composition of the major macronutrients [63]. Weight loss diets confer similar results irrespective of their fat and fatty acid composition, as shown in a study comparing very low-fat (12% energy) with a high monounsaturated-fat (35% energy) test diet. Corresponding reductions in LDLc were minor and similar [64].

### 4.6. Summarizing the Potential Reduction in LDLc by Dietary Means

The most potent dietary modification that is consistently achievable within customary patterns of eating is the substitution of 5% or more of saturated fatty acid by similar amounts of polyunsaturated fatty acid, which could lead to a 10% or more reduction in LDLc. Substituting with monounsaturated fatty acids would be somewhat less effective, but in practice both unsaturated fatty acid-enriched foods and oils would be advised. Reducing dietary cholesterol is considerably less effective but should be implemented when CVD risk is high. A large number of other foods, including nuts, brans, and supplements such as phytosterols, which have been shown to lower LDLc by around 5% if eaten in adequate amount, could be considered. What is uncertain is the extent to which combinations of these foods and supplements would produce a substantial additive benefit. One study quoted above suggests that this could lower LDLc by up to 20%, which has also been shown in compliers with healthful patterns of eating such as the Step 2 AHA diet. A 20% reduction in LDLc is at the lower end of statin-inducible LDLc lowering.

## 5. Recent Pharmacological Advances for Lowering LDLc

As an introductory note, most national regulators of therapeutics require that before lipid-lowering drugs are prescribed, a cholesterol-lowering diet should be implemented [40,65,66]. Moderate intensity statin dosage complemented by ezetimibe combines inhibition of cholesterol synthesis with halving of cholesterol absorption. LDLc reduction was about 50% in the IMPROVE-IT trial of statin (40 mg simvastatin) plus ezetimibe (10 mg), representing a 24% further lowering of LDLc compared with statin monotherapy [67]. When greater reductions are needed, as in severely affected patients with familial hypercholesterolemia, other pharmacological approaches may be needed.

In recent years, pharmacology has focused on suppressing the circulating concentration of the enzyme proprotein subtilisin/cexin type 9 (PCSK9), the function of which is to facilitate the degradation of LDL receptors [68]. Individuals who overexpress PCSK9 have high levels of plasma LDL and show increased risk for CVD. By contrast, individuals with the null mutation for PCSK9 have low LDL levels and low CVD risk [69]. Monoclonal antibodies that bind PCSK9 in plasma, such as evolocumab and arilocumab administered subcutaneously, have shown about 50% reductions in LDLc and halving of cardiovascular events in two major trials (FOURIER and ODYSSEY, respectively) [69]. Of note, a recent report suggests PCSK9 inhibition may be less effective in LDLc reduction in women than in men. A meta-analysis of 6 studies comprising 1216 men and 641 women showed that despite higher baseline LDLc levels in women, the effects of PCSK9 monoclonal antibodies on LDLc reduction were significantly greater in men than in women (mean difference = 17.4 mg/dL) [70]. Using the PCSK9-R46L loss-of-function variant as a means to examine the genetic inhibition of PCSK9 in the UK Biobank general population cohort (219,301 women and 163,512 men), the study confirmed that the decrease in LDLc associated with PCSK9-R46L was less marked in women than in men [70]. More recently, evinacumab, an inhibitor of angiopoietin-like 3 (ANGPTL3), has shown the capacity to reduce LDLc significantly, even in homozygotes for familial hypercholesterolemia [71]. ANGPTL3 is an inhibitor of lipoprotein and endothelial lipase and plays a key role in lipid metabolism by increasing the levels of triglycerides and other lipids.

Greater reductions in LDLc have been achieved by RNA-silencing technology (siRNA). Inclisiran is one such drug that inhibits hepatic production of mRNA that normally translates PCSK9 protein in the liver, the levels of which in plasma decline by approximately 80% depending on the dose and LDLc reductions of at least 50% [72]. Whereas the monoclonal inhibitors require injections every two weeks or at least monthly, the siRNA approach requires only six monthly injections. Antisense oligonucleotides targeting ANGPTL3 RNA initially developed for treating severe hypertriglyceridemia have shown substantial LDLc reduction [73]. In a randomized, double-blind, dose-escalation phase I clinical trial, healthy volunteers with elevated triglycerides and patients with familial hypercholesterolemia received subcutaneous injections of placebo or an antisense oligonucleotide targeting ANGPTL3 mRNA in a single dose or multiple doses weekly for 6 weeks [74]. There were significant reductions in absolute levels of ANGPTL3 protein (46.6–84.5%) and LDLc (up to 32.9%). There were no serious side effects observed.

There are several alternative approaches targeting the PCSK9 gene in the form of smaller molecules eventually leading to genetic manipulation, which has life-long efficacy [73].

## 6. Conclusions (Figure 1)

1. LDLc is lowered by a variety of dietary interventions that achieve as much as a 20% reduction. Adequate substitutions of saturated fatty acids by polyunsaturated fatty acids have shown, on average, about a 10% lowering. Other interventions reduce LDLc by about 5% each and include reductions in cholesterol consumption and supplementing with plant sterols and other nutrients.
2. Further research should focus on combinations of foods, specific nutrients, and supplements aiming for total LDLc reductions of 20%, which would be competitive with simple pharmacological therapies such as statins or ezetimibe. For the large population with mild-to-moderate hypercholesterolemia, successful interventions through diet are more desirable but more difficult to achieve currently than pharmacologically. Enthusiastic support from health professionals is essential. Appropriate manufacturing and pricing of foods fortified to lower LDLc is required.
3. The advent of drugs more potent than statins, such as the siRNA inhibitor of PCSK9 that lowers LDLc by >25% and requires only twice-yearly subcutaneous injections, will be competitive with diet once it becomes affordable to most patients.

**Figure 1:**
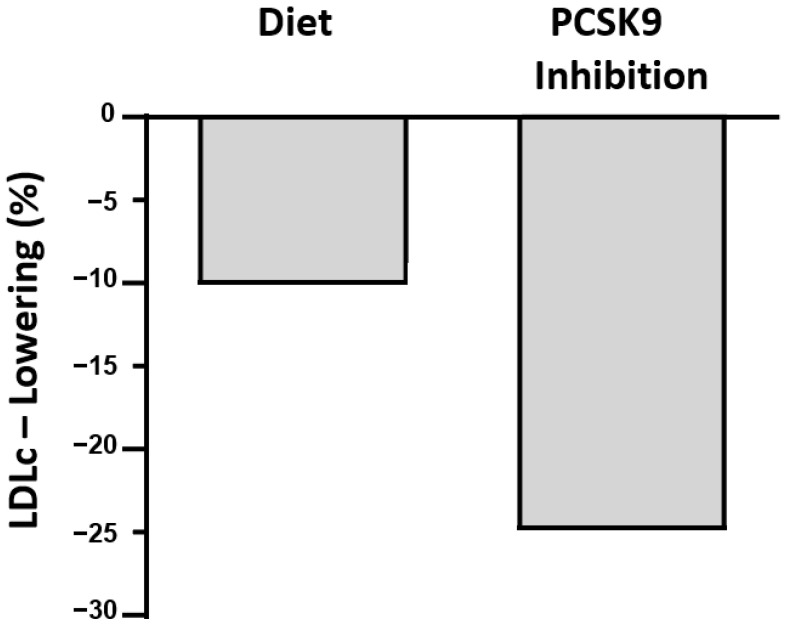
Illustrative depiction of relative LDLc lowering by diet and pharmacotherapy (as referenced in text).

## Data Availability

No new data were created or analyzed in this study. Data sharing is not applicable to this article.
